# Heparan sulfate endosulfatases, Sulf1 and Sulf2, are required for formation of the medial habenula-interpeduncular nucleus circuit

**DOI:** 10.3389/fnana.2026.1961110

**Published:** 2026-09-17

**Authors:** Sayaka Hashimoto, Takuya Okada, Kazuko Keino-Masu, Ken Miya, Masayuki Masu

**Affiliations:** 1 Graduate School of Comprehensive Human Sciences, University of Tsukuba, Tsukuba, Ibaraki, Japan; 2 Department of Molecular Neurobiology, Institute of Medicine, University of Tsukuba, Tsukuba, Japan

**Keywords:** axon guidance, habenula, interpeduncular nucleus, midline crossing, Sulfatase 1, Sulfatase 2

## Abstract

The medial habenula (MHb), a medial subdivision of the habenula located in the dorsal diencephalon, sends axons towards the interpeduncular nucleus (IPN) through the fasciculus retroflexus. Upon reaching the IPN, MHb axons cross the midline multiple times. Although heparan sulfate proteoglycans have previously been shown to play a role in MHb axon guidance, the role of heparan sulfate 6-O-endosulfatases, Sulfatase 1 (Sulf1) and Sulfatase 2 (Sulf2), has to date not been investigated. We thus examined MHb axon trajectories in *Sulf1* and *Sulf2* knockout mice and found that *Sulf1/2* double knockout (DKO) mice showed several types of pathfinding errors after reaching the ventral surface of the brain. The abnormalities included aberrant anterior extension into the mammillary body, abnormal lateral projection on the brain surface, and reduced midline crossing within the IPN. Furthermore, the DKO mice exhibited altered outcomes in social conflict in a tube test assay: they showed a higher win rate against wild-type mice. This behavioral phenotype may reflect functional impairment of the MHb-IPN circuit resulting from its developmental abnormalities. Taken together, these findings suggest that Sulf1/2 are required for proper MHb-IPN circuit formation during development, which is in turn critical for its normal function in adulthood.

## Introduction

The habenula (Hb) is an evolutionarily conserved structure located in the dorsal diencephalon. It receives inputs from the limbic forebrain and basal ganglia and sends outputs to the midbrain and hindbrain regions through the fasciculus retroflexus (Hikosaka, 2010; Aizawa et al., 2011, 2012; Hu et al., 2020). The Hb consists of the medial and lateral subnuclei (the MHb and LHb, respectively), which exhibit distinct gene expression, connectivity, and functions. The MHb axons mainly innervate the interpeduncular nucleus (IPN), whereas the LHb axons project to the monoaminergic nuclei in the midbrain and hindbrain (Hikosaka, 2010; Aizawa et al., 2011; Hu et al., 2020). The MHb-IPN circuit has been implicated in aversion, stress responses, anxiety-like behavior, depression, drug addiction, and nicotine dependence (Viswanath et al., 2014; McLaughlin et al., 2017; Lee et al., 2019; Ciscato et al., 2025). In addition, cholinergic transmission in the MHb-IPN circuit was recently shown to affect the outcome of social conflicts in male mice (Matsumata et al., 2025). Conditional knockout of choline acetyltransferase (ChAT) in the MHb increased the win rate in the tube test, a behavioral assay to evaluate social dominance (Fan et al., 2019). Conversely, optogenetic activation of MHb neurons increased the loss rate (Matsumata et al., 2025).

During formation of the fasciculus retroflexus, MHb axons grow ventrally towards the diencephalic floor plate, turn caudally to reach the IPN, and then cross the midline multiple times within the IPN (Iwahori et al., 1993; Bianco et al., 2008; Bianco and Wilson, 2009; Beretta et al., 2012; Moreno-Bravo et al., 2016). It has been shown that many axon guidance molecules regulate axonal projections from the MHb to the IPN (Funato et al., 2000; Kantor et al., 2004; Belle et al., 2014; Moreno-Bravo et al., 2016). Semaphorin 3F (Sema3F) is expressed adjacent to the developing fasciculus retroflexus and repels the MHb axons to guide them along a defined trajectory (Funato et al., 2000; Sahay et al., 2003). Sema5A, another member of the semaphorin family, is also required for MHb axon guidance (Kantor et al., 2004). Previous studies showed that Netrin-1 and its receptor DCC play roles in MHb axonal projections (Funato et al., 2000; Belle et al., 2014). Both *Ntn1* knockout (KO) mice, which encodes Netrin-1, and *Dcc* KO mice exhibit severe MHb axon guidance defects, including defasciculation, abnormal rostrocaudal projections from the MHb, and impaired midline crossing in the IPN (Belle et al., 2014). In addition, Slit-Robo signaling regulates MHb axon behavior at the ventral midline (Belle et al., 2014; Moreno-Bravo et al., 2016). In *Slit*/*Robo* mutant embryos, although MHb axons reach the IPN, they show impaired midline crossing/recrossing and abnormal rostrocaudal extension along the floor plate (Belle et al., 2014).

Heparan sulfate proteoglycans (HSPGs), which are present on the cell surface and in the extracellular matrix, regulate multiple aspects of neural development, including axon guidance (Holt and Dickson, 2005; Masu, 2016; Condomitti and de Wit, 2018; Yu et al., 2018). HSPGs interact with various signaling molecules and receptors through heparan sulfate (HS) sugar chains. Sulfation patterns of HS, generated by sulfotransferases and then remodeled by HS endosulfatases, determine the specificity and affinity of HS-protein interactions (Perrimon and Bernfield, 2000; Bishop et al., 2007; Masu, 2016; Yu et al., 2018). HS endosulfatases, Sulfatase 1 (Sulf1) and Sulfatase 2 (Sulf2), modulate diverse cellular signaling pathways either positively or negatively by removing 6-*O*-sulfate groups from mature HS chains extracellularly (Dhoot et al., 2001; Morimoto-Tomita et al., 2002; Lamanna et al., 2007; El Masri et al., 2017; Boustany et al., 2026). Our previous studies using *Sulf1/2* double knockout (DKO) mice have shown that Sulf1/2 play important roles in the formation of the corticospinal tract, corpus callosum, and dorsal hippocampal commissure (Okada et al., 2017; Aizawa et al., 2020; Tsuji et al., 2022). The action of Sema5A on MHb axons is regulated by HSPGs and chondroitin sulfate proteoglycans (CSPGs), another major class of proteoglycans (Kantor et al., 2004; Masu, 2016; Yu et al., 2018). HSPGs expressed on the surface of MHb axons are required for Sema5A-mediated attraction. By contrast, CSPGs enriched around the MHb axonal trajectory convert Sema5A from an attractive to a repulsive guidance cue (Kantor et al., 2004). Thus, although previous studies have implicated HSPGs in MHb axon guidance, the role of Sulf1/2 remains unclear.

In this study, we examined MHb axon guidance defects in *Sulf1/2* DKO mice. We found that the DKO mice show aberrant anterior and lateral extension of MHb axons and decreased midline crossing within the IPN. Furthermore, the DKO mice exhibit altered outcomes in social conflict in the tube test. Our data suggest that Sulf1/2 play a role in the proper pathfinding and midline crossing of MHb axons as well as in the functions of the MHb-IPN circuit.

## Materials and methods

### Mice

*Sulf1* and *Sulf2* KO mice were generated as described previously (Nagamine et al., 2012; Sulf1^tm1Mmas^, MGI 5318489; Sulf2^tm1Mmas^, MGI 5318490). The mouse embryos were derived from mice backcrossed to C57BL/6N for 5 generations (N5 generation; Okada et al., 2017). To analyze DKO embryos, we mated *Sulf1*^−/−^; S*ulf2*^+/−^ mice and used single *Sulf1* KO mice as controls because littermate comparison was important and because *Sulf1* KO did not affect the gross brain anatomy (Okada et al., 2017) nor MHb-IPN circuit formation (this study). In addition, this breeding scheme has the advantage of being more efficient than breeding to produce both wild-type (WT) and DKO embryos from the same litters. Noon of the day on which a vaginal plug was observed was designated as embryonic day 0.5 (E0.5). Although *Sulf1/2* DKO mice on the N5 C57BL/6N background die shortly after birth, offspring on a mixed genetic background of C57BL/6N and CD-1/ICR survive into adulthood (Aizawa et al., 2020). Therefore, adult WT and DKO mice were analyzed on this mixed background. The genotypes were determined by PCR of genomic DNAs isolated from mouse tails or embryos. All animal experiments were approved by and performed according to the guidelines of the Animal Care and Use Committee of the University of Tsukuba.

### Preparation of brain sections

Adult mice were deeply anesthetized with an overdose of pentobarbital administered intraperitoneally. E17.5 mouse embryos were similarly anesthetized after being removed from pregnant mice euthanized by cervical dislocation. Adult mice and embryos were transcardially perfused with 4% paraformaldehyde (PFA) in phosphate-buffered saline (PBS). The brains were dissected and postfixed in the same fixative overnight at 4 °C. After cryoprotection with 30% sucrose in PBS, the brains were embedded in Tissue-Tek O. C. T. Compound (Sakura Finetek Japan, Tokyo, Japan). Coronal sections of adult brains (50 μm thick) were cut by use of a cryostat (CM 1850; Leica Biosystems, Wetzlar, Germany) and collected in PBS. Coronal sections of embryonic brains (20 μm thick) were mounted on MAS-coated slide glasses (Matsunami Glass Industry, Osaka, Japan).

### Immunohistochemistry

The sections were washed in PBS with 0.1% Tween-20 (PBT), followed by stepwise dehydration through 25 to 80% methanol/PBT and then 80% methanol/20% dimethyl sulfoxide (DMSO). To block endogenous peroxidase activity, the sections were incubated in 3% H_2_O_2_/80% methanol/20% DMSO for 30 min. The sections were rehydrated and washed with PBT, and then incubated overnight with primary antibodies in 0.5% blocking reagent (Roche Diagnostics, Mannheim, Germany) in PBT. After being washed with PBT, the sections were incubated with biotin- or fluorophore-conjugated secondary antibodies diluted in the same blocking solution for 2 h. The primary antibodies used were anti-choline acetyltransferase (ChAT) (AB144P, 1:250 dilution; Merck Millipore, Darmstadt, Germany), anti-Pax7 (AB_528428, 1:50 dilution; Developmental Studies Hybridoma Bank, Iowa City, IA, USA), and anti-Robo3 (AF3076, 1:300 dilution; R&D Systems, Minneapolis, MN, USA). The secondary antibodies used were biotin-conjugated anti-goat IgG antibody (1:600 dilution; Vector Laboratories, Burlingame, CA, USA), Alexa Fluor 488-conjugated donkey anti-goat IgG (A11055, 1:1000 dilution; Molecular Probes, Eugene, OR, USA), and Cy3-conjugated donkey anti-mouse IgG (715–165-150, 1:1000 dilution; Jackson ImmunoResearch, West Grove, PA, USA). For chromogenic staining, the sections were washed with PBT, then with Tris–HCl, pH 7.6 containing 0.8% NaCl, 0.02% KCl, and 0.1% Tween-20 (0.1% Tween-20/TBS), and subsequently incubated with an avidin-biotin-peroxidase complex (ABC; Vectastain Elite ABC kit, Vector Laboratories) for 30 min. After additional washes with 1% Tween-20/TBS and then with 0.1% Tween-20/TBS, the sections were incubated with 3,3′-diaminobenzidine (DAB; Vector Laboratories) for 10 min. All steps were performed at room temperature. After staining, free-floating adult brain sections were mounted on MAS-coated glass slides (Matsunami Glass Industry). All sections were coverslipped with Fluoromount-G (Southern Biotech, Birmingham, AL, USA). Chromogenic and fluorescence images were observed and recorded by use of microscopes (Axioplan 2 and LSM510, Carl Zeiss Microscopy, Jena, Germany; and BZ-8000, Keyence, Osaka, Japan).

### Anterograde tracing of MHb fibers

Anterograde tracing with biotinylated dextran amine (BDA; 10,000 MW, lysine-fixable; Molecular Probes, Eugene, OR, USA) was performed as described previously (Aizawa et al., 2020). Adult male mice (9–23 weeks; WT, *n* = 3; DKO, *n* = 2) were anesthetized with pentobarbital (50 mg/kg body weight, intraperitoneal injection) and fixed in a stereotaxic frame (David Kopf Instruments, Tujunga, CA, USA). After scalp incision, a burr hole was made, and 0.3–0.5 μL of 10% BDA in PBS was injected into the left MHb through the burr hole by use of a pressure microinjector (KDS 101; KD Scientific, Holliston, MA, USA). The stereotaxic coordinates were as follows: anterior–posterior 1.5 mm, medial-lateral 0.6–0.7 mm, dorsal-ventral 2.6–2.7 mm with a glass capillary inserted through the right hemisphere and angled 20° medially from the vertical in the coronal plane. The mice were transcardially perfused with 4% PFA/PBS 4 days after injection. To visualize the axons of the fasciculus retroflexus, the brain sections were prepared as described above and incubated with ABC and DAB for 30 min and 10 min, respectively. After being mounted on MAS-coated glass slides (Matsunami Glass Industry), the sections were dehydrated through a graded ethanol series, cleared in xylene, and coverslipped with Poly-Mount (Polysciences, Warrington, PA, USA).

### 3D reconstruction

3D reconstruction was performed as described previously (Okada et al., 2019). The serial 2D images of the sections were aligned by use of AutoAligner alignment software (Bitplane, Zürich, Switzerland) and reconstructed into 3D images by use of Imaris software (Bitplane).

### Tube test assay

The tube test was performed as described previously (Fan et al., 2019). A total of 16 singly-housed male mice, 8 WT and 8 DKO, were used. The age in weeks of the mice at the start of the test was 22–23 for WT and 21–25 for DKO in the first batch, and 12 for WT and 10–12 for DKO in the second batch. The tests were conducted independently in 2 batches, each consisting of 4 WT and 4 DKO mice. Before testing, the mice were trained to traverse a transparent tube (3 cm in inner diameter and 30 cm in length) for 2 d, with a total of 10 times per day, 5 times from each end. During the testing stage, 2 mice were placed at opposite ends of the tube and allowed to enter simultaneously in each trial. After having been trained to move forward, the 2 mice met in the middle of the tube. Upon encountering in the central region of the tube, one of the mice gave way to the other. The mouse that forced its opponent out of the tube within 2 min was designated as the winner, whereas the other that retreated from the tube was designated as the loser. A mouse was considered to have lost when all four limbs were outside the tube. If both mice remained in the tube for 2 min, the trial was defined as a draw. In a total of 128 WT vs. DKO matches, only one resulted in a draw. Win probabilities were analyzed for the remaining 127 matches in which a winner was determined, as described below. Testing was conducted by use of a round-robin design over 8 days, with 4 matches between the same combination of mice; as a result, each mouse conducted 28 trials (3–4 trials per day). The order of trials was arranged to avoid consecutive testing of the same mouse.

### Statistical analysis

Statistical analyses for the tube test were performed by use of R version 4.4.2 with the lme4 package version 1.1–38 (Bates et al., 2015). Win-loss outcomes were analyzed with a binomial generalized linear mixed model (GLMM) to account for the binary nature of the data and repeated measurements from the same individuals. Genotype was included as a fixed effect and mouse as a random effect. Differences in body weight between the WT and DKO mice were analyzed by use of the Mann–Whitney *U* test with SPSS Statistics (Version 30.0; IBM, Armonk, NY, USA). Correlation analyses between the win rate and the body weight rank were performed by use of the Pearson correlation coefficient in Microsoft Excel (Microsoft, Redmond, WA, USA).

## Results

### Abnormal projections of cholinergic neurons in the medial habenula in the adult *Sulf1/2* DKO brain

To examine the axonal projections of the cholinergic neurons in the ventral subregions of the medial habenula (MHb), we performed immunostaining of choline acetyltransferase (ChAT). In the WT mice, the cholinergic fibers extended towards the IPN, a ventral midline structure of the midbrain, through the fasciculus retroflexus (Figures 1A_1_–F_1_,C_1_’–F_1_’). These fibers formed dense innervation within the IPN. In the DKO mice, the fasciculus retroflexus formed normally (Figures 1A_2_–B_2_), whereas some fibers were observed to deviate laterally as they approached the ventral surface of the brain (Figures 1C_2_,C_2_’, arrowheads). At the level of the rostral IPN, the fasciculus retroflexus defasciculated, and thick bundles of laterally projecting fibers were found on the brain surface, overlapping with the oculomotor nerve fibers (Figures 1D_2_–E_2_,D_2_’–E_2_’, arrowheads). These aberrant fibers remained on the brain surface near the IPN, showing no further projection. In the IPN, the distribution of the cholinergic fibers appeared slightly distorted and flattened along the dorsoventral axis (Figures 1F_2_,F_2_’). In addition to those in the IPN, aberrant fibers were also observed in the mammillary body (Figures 1D_2_,D_2_’, arrows). No abnormalities in cholinergic fiber projections were observed in either *Sulf1* or *Sulf2* single KO mice (data not shown).

**Figure 1:**
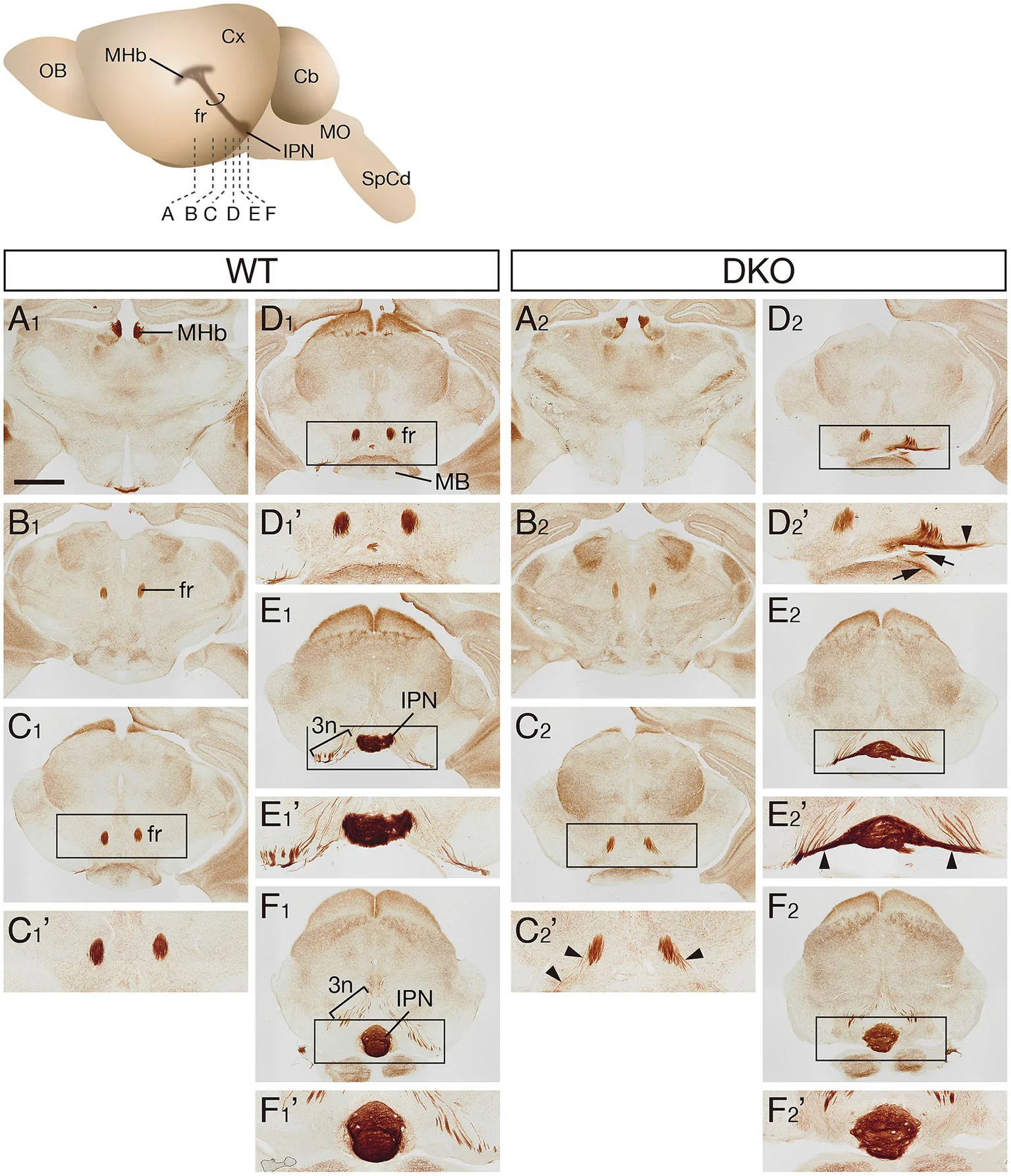
ChAT immunostaining of the adult mouse brain. **(A–F)** Coronal sections of WT **(A**_**1**_**–F**_**1**_**)** and DKO **(A**_**2**_**–F**_**2**_**)** brains are shown. The positions of sections **(A–F)** in the brain are indicated by the broken lines in the upper diagram. **(C**_**1**_**’–F**_**1**_**’)** and **(C**_**2**_**’–F**_**2**_**’)** are the magnified images of the boxed regions in **(C**_**1**_**–F**_**1**_**)** and **(C**_**2**_**–F**_**2**_**)**, respectively. In the WT brain, cholinergic neurons in the MHb **(A**_**1**_**)** formed the fasciculus retroflexus **(**fr, **B**_**1**_**–D**_**1**_**)** and projected to the IPN in the ventral midline of the midbrain **(E**_**1**_,**F**_**1**_**)**. In the DKO brain, abnormal fibers projecting laterally were observed **(C**_**2**_**’–E**_**2**_**’**, arrowheads**)**. Some aberrant axon bundles invaded the mammillary body **(D**_**2**_**’**, arrows**)**. 3n, oculomotor nerve; Cb, cerebellum; Cx, cerebral cortex; MB, mammillary body; MO, medulla oblongata; OB, olfactory bulb; SpCd, spinal cord. The scale bars indicate 1.0 mm **(A**_**1**_**–F**_**1**_,**A**_**2**_**–F**_**2**_**)** and 500 μm **(C**_**1**_**’–F**_**1**_**′**,**C**_**2**_**’–F**_**2**_**′)**.

To obtain the overall view of the fasciculus retroflexus, 3D images were reconstructed from the ChAT-stained brain sections. In the WT brain, the axons of the fasciculus retroflexus extending in the posteroventral direction from the MHb were clearly visualized (Figures 2A–C). The axons bulged slightly outward along their trajectory before projecting to the IPN (Figure 2C). The IPN appeared to be elongated along the anterodorsal-posteroventral axis (Figure 2B). In the DKO brain, although the formation of the fasciculus retroflexus appeared normal (Figures 2D–F), laterally misdirected fibers were observed on the ventral surface of the midbrain (Figure 2F, arrowheads), and the IPN was reduced in size along both the anteroposterior and the dorsoventral axes (Figures 2E,F).

**Figure 2:**
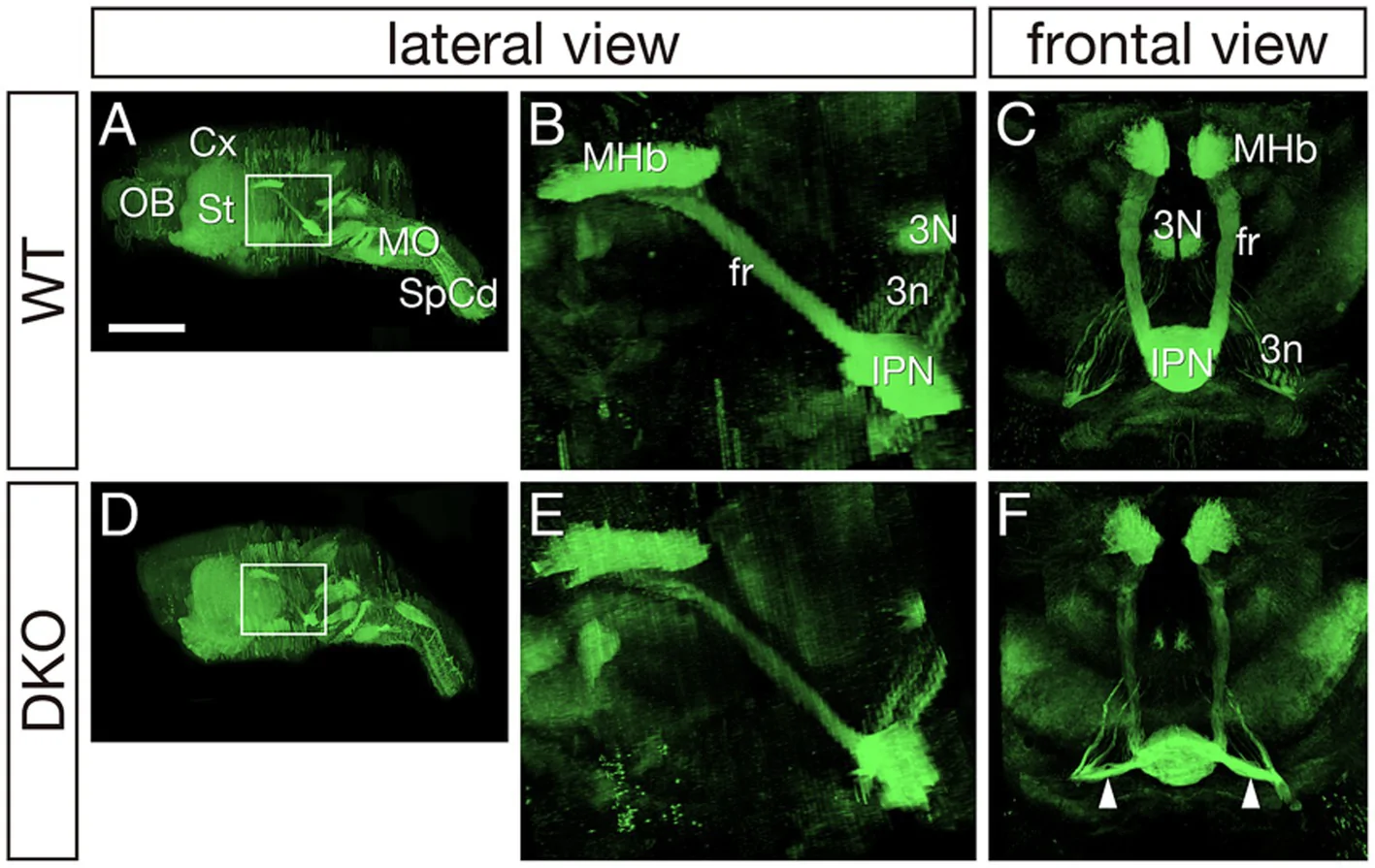
3D reconstruction of the MHb-IPN circuit. **(A–F)** 3D reconstructed images from serial coronal sections of WT **(A–C)** and DKO **(D–F)** brains immunostained with an anti-ChAT antibody are shown. Lateral **(A**,**B**,**D**,**E)** and frontal **(C**,**F)** views of the 3D images are shown. **(B**,**C)** and **(E**,**F)** are the magnified images of the boxed regions in **(A)** and **(D)**, respectively. Signals are pseudocolored in pale green. In the WT brain, thick bundles of the fasciculus retroflexus (fr) extended to the IPN **(B**,**C)**. Axons of the oculomotor nerve (3n) were observed lateral to the IPN **(C)**. In the DKO brain, abnormal fibers projected laterally on the ventral surface of the midbrain **(F**, arrowheads**)**. 3N, oculomotor nucleus; Cx, cerebral cortex; MO, medulla oblongata; OB, olfactory bulb; St, striatum; SpCd, spinal cord. The scale bars indicate 3.0 mm **(A**,**D)** and 600 μm **(B**,**C**,**E**,**F)**.

### Defects in midline crossing and recrossing of MHb fibers in the *Sulf1/2* DKO brain

To further investigate the abnormalities revealed by ChAT immunostaining, we stereotaxically injected biotinylated dextran amine (BDA) into the unilateral MHb to trace its axons (Figures 3A,A’). The BDA-labeled fibers were visualized with the avidin-biotin peroxidase complex (ABC) and 3,3′-diaminobenzidine (DAB) reaction (Figures 3B,C), followed by 3D reconstruction from serial sections (Figures 3D,E). In the WT brain, the BDA-labeled fibers extended towards the midline (Figure 3B_1_) and terminated within the IPN (Figures 3C_1_,D). In the DKO brain, the BDA-labeled fibers defasciculated just before reaching the ventral brain surface (Figure 3B_2-1_), similar to the defasciculation observed by ChAT immunostaining (Figure 1D_2_’). The BDA-labeled fibers were observed bilaterally on the brain surface (Figures 3B_2_,C_2_). Furthermore, the 3D reconstructed images clearly revealed 2 populations of abnormal fibers: midline-crossing fibers extending along the contralateral ventral brain surface (Figure 3E, open arrowheads) and uncrossed fibers extending along the ipsilateral ventral brain surface (Figure 3E, filled arrowheads). Although some BDA leakage was observed in the medial part of the contralateral cerebral cortex, which was along the injection capillary’s path, the projections originating from the labeled cortex were far from those of the MHb axons, leaving our findings unaffected.

**Figure 3:**
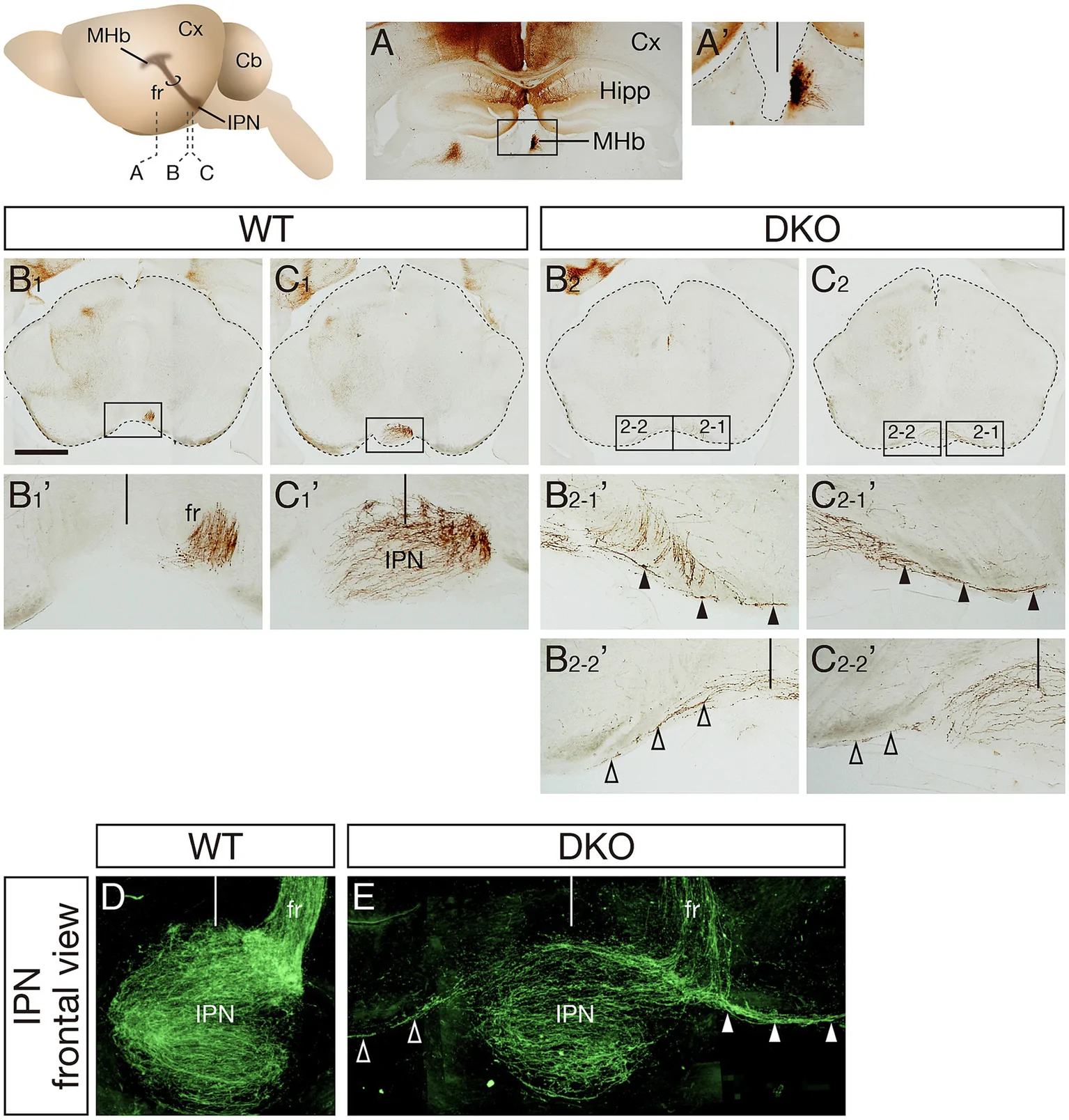
Unilateral BDA tracing of the MHb-IPN circuit. **(A)** A representative brain section of the BDA injection site in the MHb. **(A’)** is the magnified image of the boxed region in **(A)**. **(B**,**C)** Coronal sections of BDA-injected WT **(B**_**1**_,**C**_**1**_**)** and DKO **(B**_**2**_,**C**_**2**_**)** brains are shown. The positions of sections **(A)–(C)** in the brain are indicated by the broken lines in the upper diagram. **(B**_**1**_**’**,**C**_**1**_**’**,**B**_**2-1**_**′**,**B**_**2-2**_**′)**, and **(C**_**2-1**_**′**,**C**_**2-2**_**’)** are the magnified images of the boxed regions in **(B**_**1**_,**C**_**1**_,**B**_**2**_, and **C**_**2**_**)**, respectively. The broken lines indicate the contour of the brain. In the WT brain, projections of the BDA-labeled fibers were confined to the IPN **(C**_**1**_**’)**. In the DKO brain, the labeled fibers extended along the brain surfaces on both the ipsilateral **(B**_**2-1**_**′**,**C**_**2-1**_**’**, filled arrowheads**)** and the contralateral **(B**_**2-2**_**′**,**C**_**2-2**_**′**, open arrowheads**)** sides of the injection site, as well as the IPN. **(D**,**E)** Frontal views of the 3D-reconstructed images of the BDA-labeled fibers in the IPN of WT **(D)** and DKO **(E)** brains. Signals are pseudocolored in pale green. Bilateral projection of the BDA-labeled fibers was clearly observed in DKO mice **(E**, filled and open arrowheads indicate ipsilateral and contralateral axons, respectively**)**. The vertical lines in **(A’**,**B**_**1**_**’**,**C**_**1**_**’**,**B**_**2-2**_**′**,**C**_**2-2**_**′**, **D**, and **E)** indicate the midline. Cb, cerebellum; Cx, cerebral cortex; fr, fasciculus retroflexus; Hipp, hippocampus. The scale bars indicate 1.0 mm **(A**,**B**_**1**_,**B**_**2**_,**C**_**1**_,**C**_**2**_**)**, 350 μm **(A’)**, and 225 μm **(B**_**1**_**′**,**C**_**1**_**’**,**B**_**2-1**_**’**,**B**_**2-2**_**′**,**C**_**2-1**_**′**,**C**_**2-2**_**′**,**D**,**E)**.

We next examined the midline crossing patterns in embryonic brains because the density of MHb fibers within the IPN was too high in the adult brain to assess the pattern of midline crossings. To this end, we performed immunostaining for Robo3, a marker of developing MHb axons (Quina et al., 2009; Belle et al., 2014; Schmidt et al., 2014). Robo3 immunostaining has previously been used to clearly visualize repeated midline crossing and to evaluate alterations in their crossing patterns in mutants with MHb axon guidance defects (Belle et al., 2014). In embryonic studies, we used mating of *Sulf1*^−/−^; S*ulf2*^+/−^ mice to obtain DKO embryos, and *Sulf1* single KO mice were used as controls because littermate comparison is important and *Sulf1* KO embryos showed normal gross brain morphology and MHb axonal projections. 3D reconstructed images of the MHb fibers in the *Sulf1* KO embryos revealed that there were 3–4 sites where MHb fibers crossed the midline in bundles (Figures 4A,B). By contrast, in the DKO mice, although some MHb fibers reached the IPN normally, there were only 1–2 sites of midline crossing within the IPN (Figure 4D). Furthermore, DKO embryos showed abnormal MHb fibers in the mammillary body (Figure 4C, arrows) and along the ventral surface of the midbrain (Figure 4D, arrowheads), similarly to those observed in adult DKO mice.

**Figure 4:**
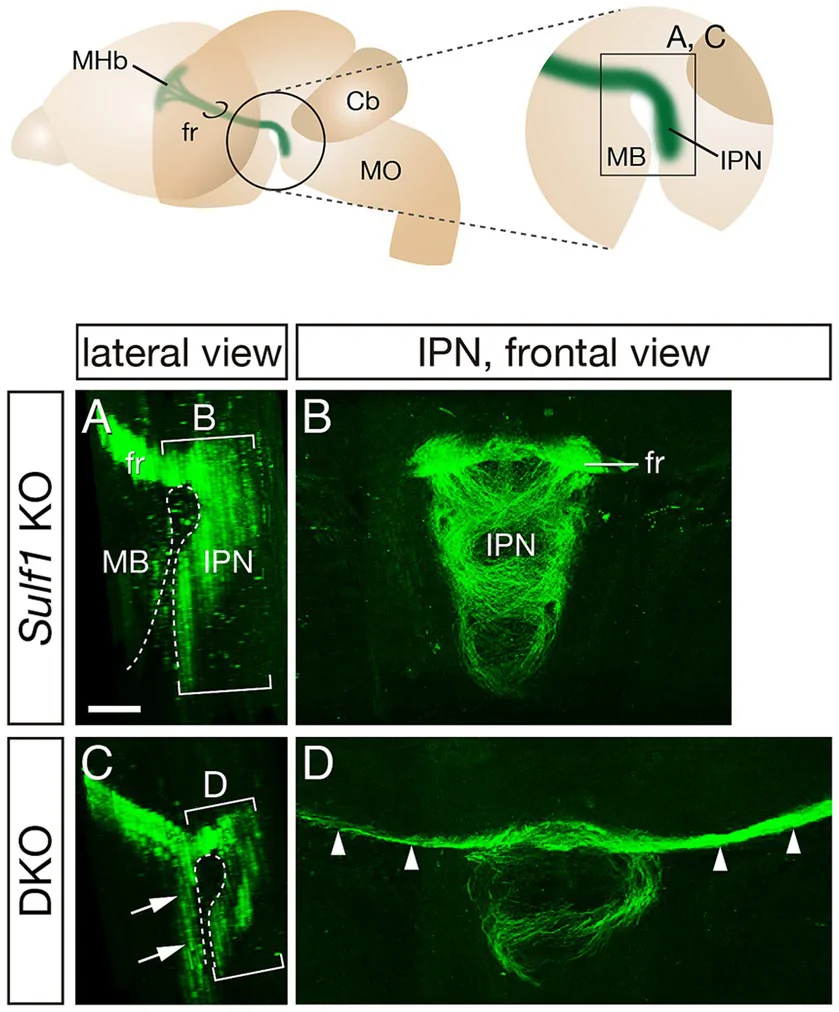
3D reconstruction of the MHb-IPN fibers within the IPN of the embryonic mouse brain. **(A–D)** 3D reconstructed images of Robo3-positive fibers in E17.5 embryos. Lateral **(A**,**C)** and frontal **(B**,**D)** views of *Sulf1* KO mice used as the control **(A**,**B)** and of DKO mice **(C**,**D)** are shown. The positions of sections **(A)** and **(C)** in the brain are indicated by the box in the upper diagram. The images **(B)** and **(D)** correspond to the areas enclosed by the brackets in **(A)** and **(C)**, respectively. The broken lines indicate the contour of the brain. In the DKO brain, Robo3-positive fibers aberrantly extended into the mammillary body (MB; **C**, arrows), whereas no such fibers were observed in the mammillary body of *Sulf1* KO embryos. **(D)** Abnormal fibers projecting laterally along the ventral surface of the brain were observed (arrowheads). Midline crossing within the IPN was also altered. Cb, cerebellum; fr, fasciculus retroflexus; MO, medulla oblongata. The scale bar indicates 200 μm.

### Ectopic localization of IPN neurons in the *Sulf1/2* DKO brain

Because ChAT immunostaining revealed an abnormal morphology of the IPN (Figures 2E,F), we next examined whether the distribution of the IPN neurons was also altered in the DKO brain. We performed double immunostaining for ChAT and paired box 7 (Pax7), an IPN neuron marker (Lorente-Cánovas et al., 2012; Moreno-Bravo et al., 2014), in adult brains. In the WT brain, the Pax7-positive neurons were confined to the IPN (Figures 5A_2_,A_3_) and not observed outside the IPN (Figure 5A_1_). By contrast, Pax7-positive neurons were ectopically localized in the DKO mice. Whereas many Pax7-positive neurons were observed in the IPN (Figures 5B_2_,B_3_), some were located outside the nucleus and positioned along the brain surface (Figure 5B_1_). Furthermore, aberrantly distributed Pax7-positive neurons were found to overlap with misdirected MHb fibers (Figure 5B_1_).

**Figure 5:**
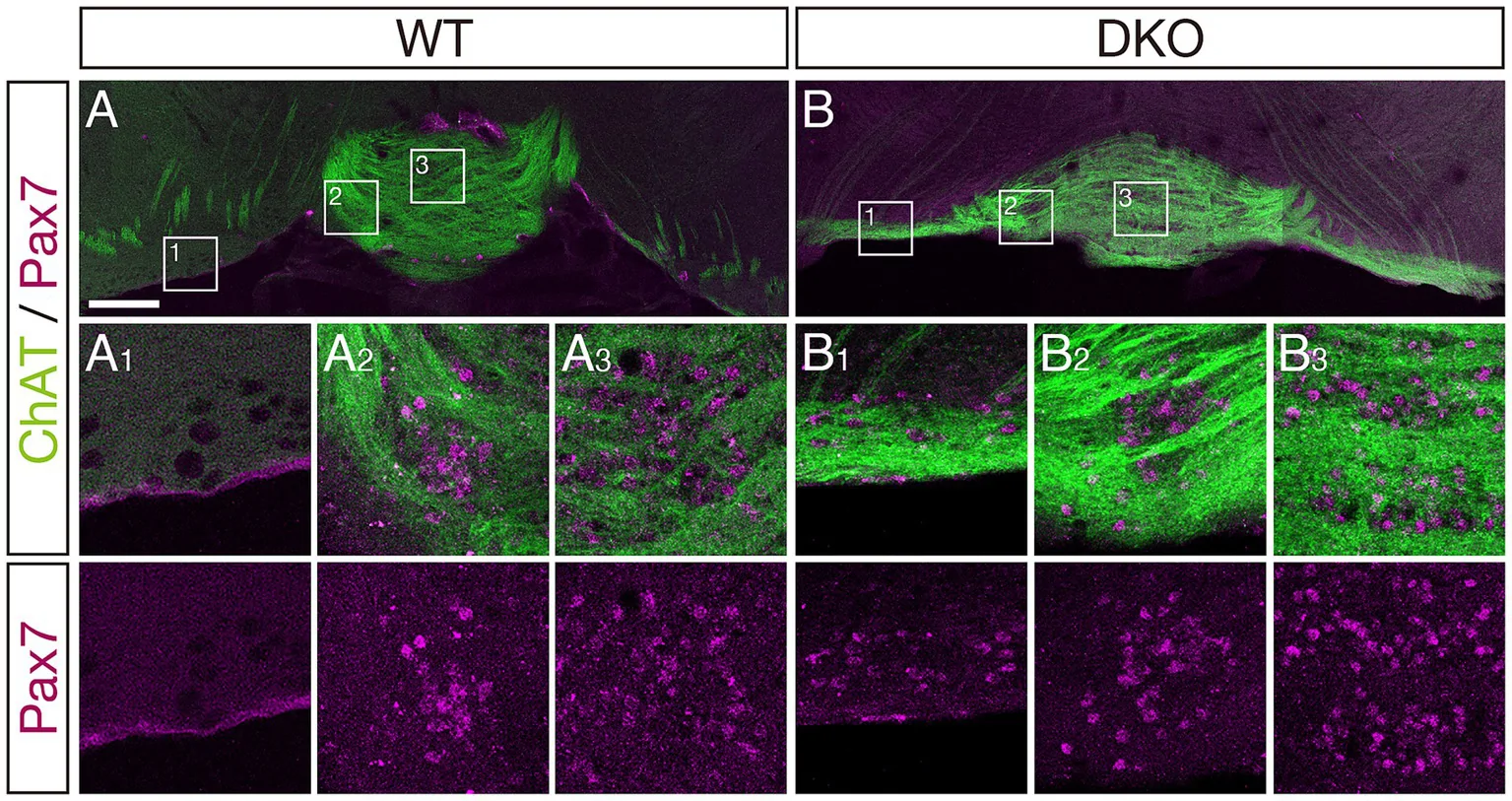
Distribution of Pax7-positive IPN neurons. **(A**,**B)** Double immunostaining for ChAT and Pax7 in coronal sections of the IPN of adult WT **(A)** and DKO **(B)** mouse brains. The signals for ChAT (green) and Pax7 (magenta) are shown. **(A**_**1**_**–A**_**3**_**)** and **(B**_**1**_**–B**_**3**_**)** are the magnified images of the boxed regions in **(A)** and **(B)**, respectively. In the DKO brain, Pax7-positive IPN neurons were ectopically localized along the misdirected ChAT-positive fibers **(B**_**1**_**)**. The signal observed along the brain surface represents nonspecific immunostaining and is unrelated to Pax7-positive IPN neurons. The scale bars indicate 200 μm **(A**,**B)**, 50 μm **(A**_**1**_**–A**_**3**_,**B**_**1**_**–B**_**3**_**)**.

### Altered social outcomes of *Sulf1/2* DKO mice in the tube test assay

Previous studies in zebrafish and mice have shown that the habenula-IPN pathway promotes losing behavior during social conflict, whereas its disruption reduces the tendency to lose (Chou et al., 2016; Kinoshita and Okamoto, 2023; Matsumata et al., 2025). Because the MHb-IPN pathway fails to form properly in the DKO mice, we wondered whether there were any changes in the tendency to lose in social conflict. To test this possibility, we performed the tube test using 8 WT and 8 DKO male mice. In 2 independent batches, contests among 4 WT and 4 DKO mice were conducted between all pairs of mice, with each pair of mice competing 4 times. Two mice were simultaneously released from the opposite ends of a transparent tube in each trial. The mouse that forced its opponent out of the tube within 2 min was defined as the winner, whereas the other that was pushed out was defined as the loser (Figure 6A). In WT vs. DKO matches, win probability was significantly higher in DKO mice than in WT mice (WT: 0.179, 95% CI [0.0662, 0.402]; DKO: 0.857, 95% CI [0.645, 0.952]; Figure 6B). Thus, DKO mice showed a reduced tendency to lose in the tube test, consistent with functional impairment resulting from aberrant MHb-IPN circuit formation. Mouse behavior in the tube test can be classified into several components, including pushing, retreating, and resisting (Zhou et al., 2017). On the basis of our qualitative observations, DKO mice appeared to persist in resisting rather than pushing back the opponents actively when encountering them face-to-face inside the tube. Their reduced tendency to lose was therefore not accompanied by more aggressive behavior. We also examined body weight, which was significantly lower in DKO mice than in WT mice (WT: 33.2 g, 95% CI [27.6, 38.8]; DKO: 24.2 g, 95% CI [21.8, 26.5]; Figure 6C). To assess whether body weight was associated with winning, we examined the relationship between body weight rank and win rate in same-genotype matches. No significant correlation was observed (Figure 6C), suggesting that differences in win probability were unlikely to be solely attributable to body weight.

**Figure 6:**
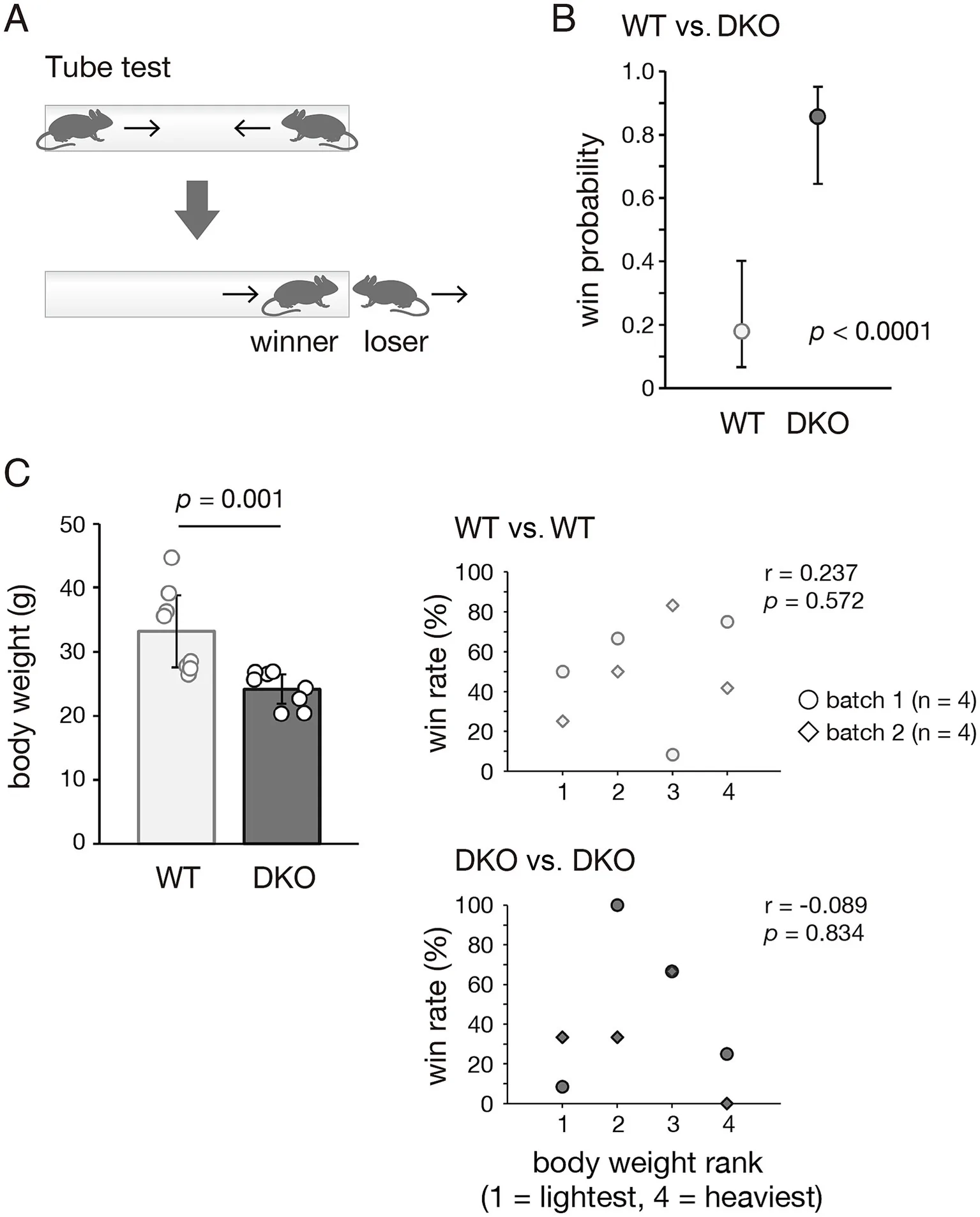
Altered outcomes in social conflict of *Sulf1/2* DKO mice in a tube test assay. **(A)** Scheme of the tube test. Two mice are placed into a transparent tube from opposite ends. When they meet in the central region of the tube, they compete to move forward. The mouse that pushes its opponent out of the tube is defined as the winner, whereas the other that retreats out of the tube is defined as the loser. **(B)** Results of the tube test. When WT and DKO mice were paired in the tube test, win probability was significantly higher in DKO mice (*n* = 8) than in WT mice (*n* = 8) (binomial generalized linear mixed model (GLMM) with genotype as a fixed effect and mouse as a random effect, *p* < 0.0001). Predicted win probability is shown with 95% confidence intervals (CIs). **(C)** (Left) Body weight on the final day of the tube test was significantly lower in DKO mice than in WT mice (Mann–Whitney *U* test, *U* = 3, *p* = 0.001). Means ± 95% CIs are shown. (Right) Scatterplots showing the relationship between win rate and body weight rank in WT–WT and DKO–DKO matches. Each genotype was tested in 2 batches of 4 mice; batch 1 and batch 2 are indicated by circles and diamonds, respectively. Pearson correlation coefficients (*r*) and *p* values are shown; no significant correlations were observed.

## Discussion

In this study, we showed that *Sulf1/2* play important roles in the formation of the neural circuit from the MHb to the IPN. In the DKO mice, the fasciculus retroflexus formed normally, indicating that *Sulf1/2* are not required for the HSPG-dependent attraction of the MHb axons by Sema5A (Kantor et al., 2004). However, 3 marked abnormalities of the MHb fibers were observed in the ventral brain regions: aberrant invasion into the mammillary body, lateral extension along the surface of the midbrain, and abnormal midline crossing within the IPN. When the fasciculus retroflexus forms approximately between E13.5 and E16.5, *Sulf1/2* mRNAs are co-expressed in radial glial cells along the ventricular zone of the third ventricle and aqueduct (Okada et al., 2017). As a result, Sulf1/2 proteins are transported to the ventral surface of the brain by the radial glial cells (Okada et al., 2017). Indeed, both Sulf1 and Sulf2 proteins were localized on the ventral brain surface, where MHb axon guidance defects were pronounced (data not shown). In addition, we have previously shown that DKO mice exhibit midline crossing defects in the pyramidal decussation, corpus callosum, and dorsal hippocampal commissure (Aizawa et al., 2020; Tsuji et al., 2022). Given that DKO mice have various abnormalities in commissural fiber formation, Sulf1/2 may play a broader role in regulating midline crossing.

In zebrafish, the dorsal Hb (dHb)-IPN pathway, homologous to the mammalian MHb-IPN pathway, plays a critical role in the resolution of social conflict, and defeat potentiates a loser-associated dHb-IPN transmission that may underlie the behavioral transition from continued fighting to submission, thereby limiting further conflict and the risk of injury (Chou et al., 2016; Kinoshita and Okamoto, 2023). A corresponding role of the MHb-IPN pathway in promoting losing behavior has also been demonstrated in mice: inhibition of its cholinergic transmission confers resistance to defeat, whereas its activation makes mice prone to defeat (Matsumata et al., 2025). Thus, the higher win probability of DKO mice observed in the present study can be interpreted as the impairment of the defeat reaction as a consequence of aberrant development of the MHb-IPN circuit in the DKO mice. Although the behavioral basis of this phenotype remains unclear, our observation that DKO mice tended to persist in resisting rather than actively pushing in the tube test is consistent with the view that a high winning probability does not necessarily indicate high levels of competitive ability.

Among various commissural fibers, MHb axons are distinctive in that they cross the midline multiple times (Iwahori et al., 1993; Bianco et al., 2008; Bianco and Wilson, 2009; O'Donnell et al., 2009; Evans and Bashaw, 2010; Beretta et al., 2012; Moreno-Bravo et al., 2016; Chédotal, 2019). The behavioral alterations of DKO mice during social conflict may result from disrupted MHb-IPN connectivity caused by axonal projection defects. In particular, impaired midline crossing and reduced axonal innervation are likely to alter the distribution or density of synaptic inputs to the IPN. Because MHb axons form en passant synapses with multiple dendrites along their trajectories within the IPN (Lenn, 1976; Hamill and Lenn, 1983), the axonal defects in DKO mice may restrict the extent of their synaptic contacts with IPN neurons. These findings may therefore provide insight into the functional significance of the repeated midline crossing of MHb axons within the IPN.

The MHb axons extend in the posteroventral direction and reach the ventral surface of the midbrain at approximately E13.5 and cross the midline in the IPN at approximately E16.5 (Belle et al., 2014; Moreno-Bravo et al., 2016). By contrast, the IPN neurons originate from the ventricular zone of the hindbrain, begin anterior migration at approximately E13.5, and complete the formation of the nucleus by approximately E18.5 (Moreno-Bravo et al., 2014). In E15.5 DKO embryos, the Robo3-positive axons already exhibited abnormal lateral extension along the ventral brain surface, whereas the Pax7-positive neurons were still migrating through the hindbrain (data not shown). Thus, the MHb axonal defects are unlikely to be caused by secondary effects of abnormal IPN neuron distribution in DKO brains. Given this possibility, the abnormal distribution of the Pax7-positive neurons along the aberrant ChAT-positive axons in the adult DKO brains may be the result of independent defects in axon guidance and neuronal migration. Alternatively, MHb axons may influence the destination of migrating IPN neurons, because previous studies have shown that axons can guide migrating neurons and thereby influence their final positions (McManus et al., 2004; Laumonnerie et al., 2015). Indeed, multiple mouse mutants with commissural axon guidance defects showed abnormal positioning of interneurons in the developing spinal cord (Laumonnerie et al., 2015). Thus, it is possible that in DKO brains, the abnormal trajectory of MHb axons alters the final destination of IPN neurons.

Previously, we reported that *Sulf1/2* DKO mice exhibit aberrant development of the corticospinal tract, corpus callosum, and dorsal hippocampal commissure (Okada et al., 2017; Aizawa et al., 2020; Tsuji et al., 2022). Notably, the corticospinal axon guidance defects are observed on the surface of the midbrain, similar to the defects of MHb axons identified in this study, suggesting that both abnormalities arise from a common mechanism. In our previous study, we demonstrated that the corticospinal axon guidance defects in the DKO brains result from Slit2-mediated repulsion (Okada et al., 2017). *Slit2* mRNA is also expressed in radial glial cells along the ventricular zone of the third ventricle and aqueduct, and Sulf1/2 and Slit2 proteins are co-localized on the ventral brain surface. Furthermore, Slit2 binds strongly to 6-*O*-sulfate groups of HS chains (Shipp and Hsieh-Wilson, 2007). Therefore, in the DKO brains, hypersulfated HS caused abnormal accumulation of Slit2 protein on the pial surface of the cerebral peduncle and hypothalamus, thereby repelling the corticospinal axons dorsally (Okada et al., 2017). Given that Slit-Robo signaling plays a critical role in MHb axon guidance (Belle et al., 2014; Moreno-Bravo et al., 2016), abnormal Slit2 accumulation may have caused aberrant repulsion of the MHb axons, leading to their abnormal extension in DKO mice. However, our proteomic analysis identified several axon guidance molecules whose abundance had changed in E15.5 DKO brains when compared with in WT brains, including Slit2 (Okada et al., 2017). Therefore, the possibility that some of these molecules other than Slit2 contributed to the abnormal projections of MHb axons should also be considered. In future studies, expression analysis of candidate molecules around the IPN and their receptors in MHb axons, as well as overexpression experiments by use of in utero electroporation, may reveal the molecular mechanisms regulating the midline crossing of MHb axons.

## Data Availability

The original contributions presented in the study are included in the article/supplementary material, further inquiries can be directed to the corresponding author.

## Glossary

ABC: avidin-biotin-peroxidase complex
BDA: biotinylated dextran amine
Cb: cerebellum
ChAT: choline acetyltransferase
CI: confidence interval
CSPG: chondroitin sulfate proteoglycan
Cx: cerebral cortex
DAB: 3,3′-diaminobenzidine
DKO: double knockout
DMSO: dimethyl sulfoxide
E: embryonic day
fr: fasciculus retroflexus
GLMM: generalized linear mixed model
Hb: habenula
Hipp: hippocampus
HS: heparan sulfate
HSPG: heparan sulfate proteoglycan
IPN: interpeduncular nucleus
KO: knockout
LHb: lateral habenula
MB: mammillary body
MHb: medial habenula
MO: medulla oblongata
OB: olfactory bulb
PBS: phosphate-buffered saline
PBT: phosphate-buffered saline with 0.1% Tween-20
PFA: paraformaldehyde
St: striatum
SpCd: spinal cord
TBS: Tris-buffered saline
WT: wild-type
3n: oculomotor nerve
3N: oculomotor nucleus
